# Zein-in-agarose microplate turbidimetric assay for screening and quantifying protease activity

**DOI:** 10.1016/j.mex.2026.104221

**Published:** 2026-10-19

**Authors:** Nenad Drulović, Luka Blagojević, Aleksandra Margetić, Nataša Božić, Biljana Dojnov, Zoran Vujčić, Marinela Šokarda Slavić

**Affiliations:** a University of Belgrade – Institute of Chemistry, Technology and Metallurgy – National Institute of the Republic of Serbia, Department of Chemistry, Belgrade, Republic of Serbia; b University of Belgrade – Innovation Centre of the Faculty of Chemistry, Belgrade, Republic of Serbia; c University of Belgrade – Faculty of Chemistry, Department of Biochemistry, Belgrade, Republic of Serbia

**Keywords:** Subtilisin, Insoluble substrate, Precipitation, High-throughput screening, Microbial proteases, Z′-factor

## Abstract

Proteolytic (zeinase) activity is most often screened on substrate-containing agar plates, where activity is scored from the diameter of a cleared halo after the residual protein has been precipitated or stained. This format is slow, semi-quantitative, subjective and low throughput. Here, we describe a miniaturised turbidimetric assay that transfers the substrate into a 96-well microplate. A zein–agarose gel is cast in each well; after the sample is added and incubated, dilute acetic acid precipitates the unhydrolysed zein, and activity is read as the increase in well transmittance at 510 nm relative to a substrate blank. The detection wavelength was selected from spectral scans, and the three gel parameters governing the signal window (zein concentration, agarose concentration and gel volume per well) were optimised by a Box–Behnken response-surface design. The optimised assay is quantitative over 1–10 mU, and its screening performance was confirmed by the Z′-factor, intra- and inter-day coefficients of variation, and limits of detection and quantification.

• 96 samples are quantified in parallel from a single plate-reader measurement.
• A continuous, objective transmittance signal replaces manual halo-diameter scoring.
• The assay is validated to high-throughput screening standards (Z′-factor, %CV, LOD/LOQ).

## Graphical abstract

**Figure:**
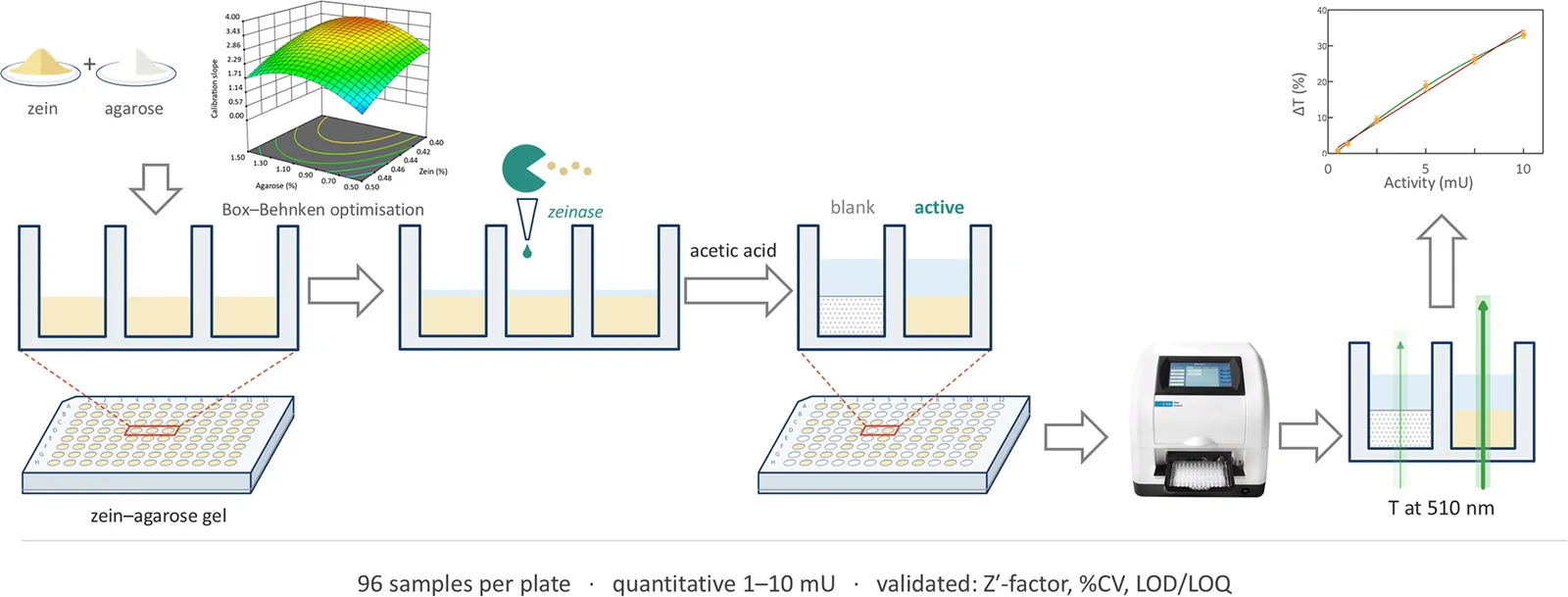

Specifications table

**Table:** 

Subject area	Biochemistry, Genetics and Molecular Biology
More specific subject area	Enzymology; protease/zeinase activity assays; high-throughput enzyme screening
Name of your method	ZIMP (Zein-In-agarose Microplate Protease assay)
Name and reference of original method	Name: Radial diffusion assay References: N.U. Mokashe, U. Patil, Quantitative protease assay by substrate-agarose plate method, J. Microbiol. Biotechnol. Food Sci. 6 (2016) 791–793. https://doi.org/10.15414/jmbfs.2016.6.2.791-793
Resource availability	Data available upon request

## Background

Proteases (peptidases, EC 3.4) account, by value for the largest single share of the global enzyme market [1]. They are central to detergents, dairy, baking and other food processing, leather tanning, and pharmaceuticals, and they are increasingly used to upgrade protein-rich waste streams [1]. Zein, the principal prolamin of maize and a major corn-milling coproduct, can be enzymatically hydrolysed into bioactive peptides, and this valorisation depends on proteases that act efficiently on native zein. Progress along this route rests on the discovery and engineering of zein-degrading proteases (zeinases), and both activities are governed by the assay used to score activity. A strain collection or a library of mutant clones can contain tens of thousands of candidates, so the assay must process large numbers of samples quickly while still returning a defensible signal of statistical quality [2,3]. The assay is therefore the practical bottleneck in moving from candidate enzyme to application.

Most protease assays rely on soluble surrogates such as casein or azocasein, which are cheap, soluble and easy to quantify. Because proteolytic activity is substrate-dependent, activity scored on these surrogates does not necessarily predict performance on native zein [4]. Zein is highly water-insoluble, which complicates measurement, but this insoluble form is what must be hydrolysed during bioprocessing; retaining native zein keeps the readout relevant to the application [5].

Clearing-zone assays, in which proteolysis creates a halo in a protein-containing agar(ose) gel, remain the conventional screen for activity on insoluble proteins such as gluten [6]. They are inexpensive and undemanding, and can be made quantitative [7], but they are low throughput and offer limited resolution; at a fixed incubation time, halo diameter increases linearly with the logarithm of enzyme activity [8]. Wells are prepared individually, halos develop over hours to days, and activity is inferred from halo diameter measured manually or by software [7,9]. Halo size also depends on enzyme diffusion, gel composition and thickness, incubation time and operator handling, limiting reproducibility between experiments. No simple, rapid, quantitative high-throughput assay currently measures proteolytic activity directly on native zein. We developed the Zein-in-agarose Microplate Protease (ZIMP) assay, which adapts the clearing-zone principle to a 96-well format in which activity is measured on native, unlabelled zein rather than on synthetic peptides or labelled derivatives. Each well contains a zein–agarose gel; after the enzyme has acted, unhydrolysed zein is precipitated with acetic acid, and activity is read as transmittance at 510 nm, which reflects hydrolysis-dependent changes in substrate turbidity relative to a blank. The format uses readily available reagents and requires no chromogenic or fluorescent probes or image acquisition,and can be implemented in any laboratory with a standard microplate reader. With hydrolysis at 40°C followed by acetic-acid precipitation, the complete procedure takes about 45 min and 96 samples are processed per plate. Assay conditions (zein concentration, agarose concentration and gel volume per well) were established by response surface methodology (Box–Behnken design). Compared with existing approaches, ZIMP differs in four respects: (i) unlike soluble-surrogate assays (casein, azocasein) and substrate-independent probe assays [4], it measures hydrolysis of native, insoluble zein, the substrate actually processed in corn valorisation; (ii) unlike clearing-zone plates [[6], [7], [8]], the readout is a continuous instrumental signal from all 96 wells in a single measurement, eliminating halo measurement and dependence on diffusion time; (iii) unlike most protease screens, it is statistically validated to high-throughput standards (Z′ = 0.74 ± 0.06, intra-/inter-day CV, LOD/LOQ) after response-surface optimisation of the gel; and (iv) it includes a defined blanking strategy for pigmented crude samples and a tunable quantification window via incubation time.

## Method details

### Reagents and solutions

● Agarose, Standard EEO grade (SERVA), used to cast the substrate gel matrix,
● Tris (Fisher Scientific),
● NaOH and HCl (Merck/Sigma-Aldrich), for pH adjustment of the Tris buffer to pH 8.5,
● Zein, purified grade (Acros Organics), substrate protein,
● SDS (sodium dodecyl sulfate, Sigma-Aldrich),
● Glacial acetic acid (Merck/Sigma-Aldrich),
● Savinase® 16L (Novozymes®), commercial subtilisin used as the reference protease for calibration. Activity of the stock was determined by the Anson–casein/Folin–Ciocalteu assay [10] (1095 U mL⁻¹, where 1 U releases 1 µmol tyrosine equivalents min⁻¹; 1 mU = 1 nmol tyrosine equivalents min⁻¹).

All reagents were of analytical grade unless otherwise stated.

### Materials

● 96-well microplates (Sarstedt), used for the assay.
● Single- and repeater pipette tips, various sizes (Eppendorf), for reagent and sample dosage.

### Instrumentation/apparatus

● Synergy LX microplate reader (BioTek/Agilent), for absorbance/transmittance reading of the 96-well plates; single-wavelength reading at 510 nm for routine assays.
● Shimadzu AX200 analytical balance, for weighing of reagents.
● inoLab pH 7110 pH meter, for buffer pH adjustment.
● Eppendorf MiniSpin Plus centrifuge.
● Eppendorf single- and repeater pipettes and a Socorex multichannel pipette, for liquid handling.
● Memmert SNB 300 thermostat, for incubation of the assay plates at 40°C.
● Lab Companion BW-20H water bath, for equilibration of the substrate gel mixture before dispensing.

### Statistical analysis

Response-surface optimisation was performed in Design-Expert 11 (Stat-Ease Inc.). All other statistical analyses were performed in JASP (v0.97.1; JASP Team) and GraphPad Prism (v8.0.2; GraphPad Software). Unless otherwise stated, data are reported as mean ± standard deviation, replicate numbers are given in the relevant tables and Fig. legends, and significance was assessed at α = 0.05.

### Protocol

A) Preparation of the zein–agarose substrate gel

1) Prepare a 10 × zein stock suspension (4.6% w/v) by dispersing the required amount of zein in 70% (v/v) ethanol.
  *Note: prepare this suspension at least one day before use to ensure complete zein solubilisation and a stable, homogeneous suspension.*
2) Dissolve agarose in 50 mM Tris–HCl buffer (pH 8.5) using 88% of the final substrate volume to final concentration of 1.21% (w/v). Heat the suspension in a microwave oven until boiling to ensure complete dissolution of the agarose.
3) Add a 10% (w/v) SDS solution at 2% of the final volume, giving a final SDS concentration of 0.2% (w/v).
4) Cool the agarose solution to approximately 70°C, then add the 10 × zein stock suspension with gentle stirring to obtain a homogeneous mixture containing 1.21% (w/v) agarose, 0.46% (w/v) zein, and 0.2% (w/v) SDS.
  *Note: These are the exact Box–Behnken-optimised values (substrate optimisation), and were confirmed experimentally; the zein is added as a 10 × (4.6% w/v) stock to reduce final ethanol concentration to 7%.*
  *Note: Once a substrate batch is prepared it can be kept at room temperature for several weeks and reheated in microwave when needed.*
5) Dispense 125 µL aliquots of the mixture into the wells of a 96-well microplate and allow the gel to solidify at room temperature for at least 10 min.
  *Note: Dispense onto a level surface and avoid disturbing the plate during solidification.*

B) Assay procedure

1) Add 20 µL of the enzyme sample to an individual well containing the solidified zein–agarose substrate gel.
2) Incubate the plate at 40°C for 25 min to allow enzymatic hydrolysis of the substrate.
  *Note: the 25 min setting provides a balanced quantification window over 1–10 mU; shorter (20 min) or longer (30–40 min) incubation shifts the sensitivity and the quantification range (see Supplementary materials).*
3) Terminate the reaction by adding 100 µL of 20% (v/v) acetic acid to each well.
4) Incubate the plate at room temperature (25°C) for 20 min to allow precipitation of the unhydrolysed zein.
5) Measure absorbance directly in the assay wells at 510 nm using a microplate reader, without removing the acetic acid, and convert absorbance (A) to transmittance (T):
  (1) $$T\left(\%\right)=10^{\left(2-A\right)}$$

C) Calibration and calculation of activity

1) On the same plate or on the same day, include a dilution series of the reference protease (we used Savinase® 16L) delivering defined activities (1–10 mU) per 20 µL, together with matched blanks (heat-inactivated sample, 15 min in boiling water) and/or a buffer blank.
2) Process the calibration wells through the same hydrolysis, stop, and development steps as the samples.
3) Calculate the blank-corrected signal for every well as
  (2) $$\Delta T\;=\;\mu T_{active}-\;\mu T_{blank}$$
  where µ is the arithmetic mean of replicate measurements.
4) Fit a second order polynomial function
  (3) $$\Delta T\;=\;a_{0}+\;a_{1}x\;+\;a_{2}x²$$
  where x is activity expressed in appropriate units, to the calibration standards.
  *Note: calibration needs to be performed for each new batch of substrate.*
5) Interpolate the activity of unknown samples from the quadratic calibration curve.

## Method validation

**Selection of the reading wavelength.** A dilution series of Savinase® 16L (Novozymes®) was prepared to deliver 1, 5 and 10 mU of proteolytic activity per 20 µL of sample, and applied to substrate gel (1.0% w/v agarose, 0.5% w/v zein, 0.2% w/v SDS, 100 µL per well) and developed under standard conditions (25 min, 40°C; acetic acid stop), alongside matched heat-inactivated and buffer blanks, and scanned from 300–800 nm (Synergy LX plate reader, sweep read, 100 ms delay, one measurement per data point; 300–800 nm in 10 nm steps). Transmittance of the active wells increased with both activity and wavelength, consistent with progressive clearing of the substrate and the wavelength-dependence of light scattering (Fig. 1). Replicate precision (n=12) was high throughout: the coefficient of variation of the active wells was ≤ 4.1 % at all wavelengths ≥ 400 nm and fell below 3 % across the mid-to-long-visible region, indicating a stable, reproducible signal.

**Fig. 1:**
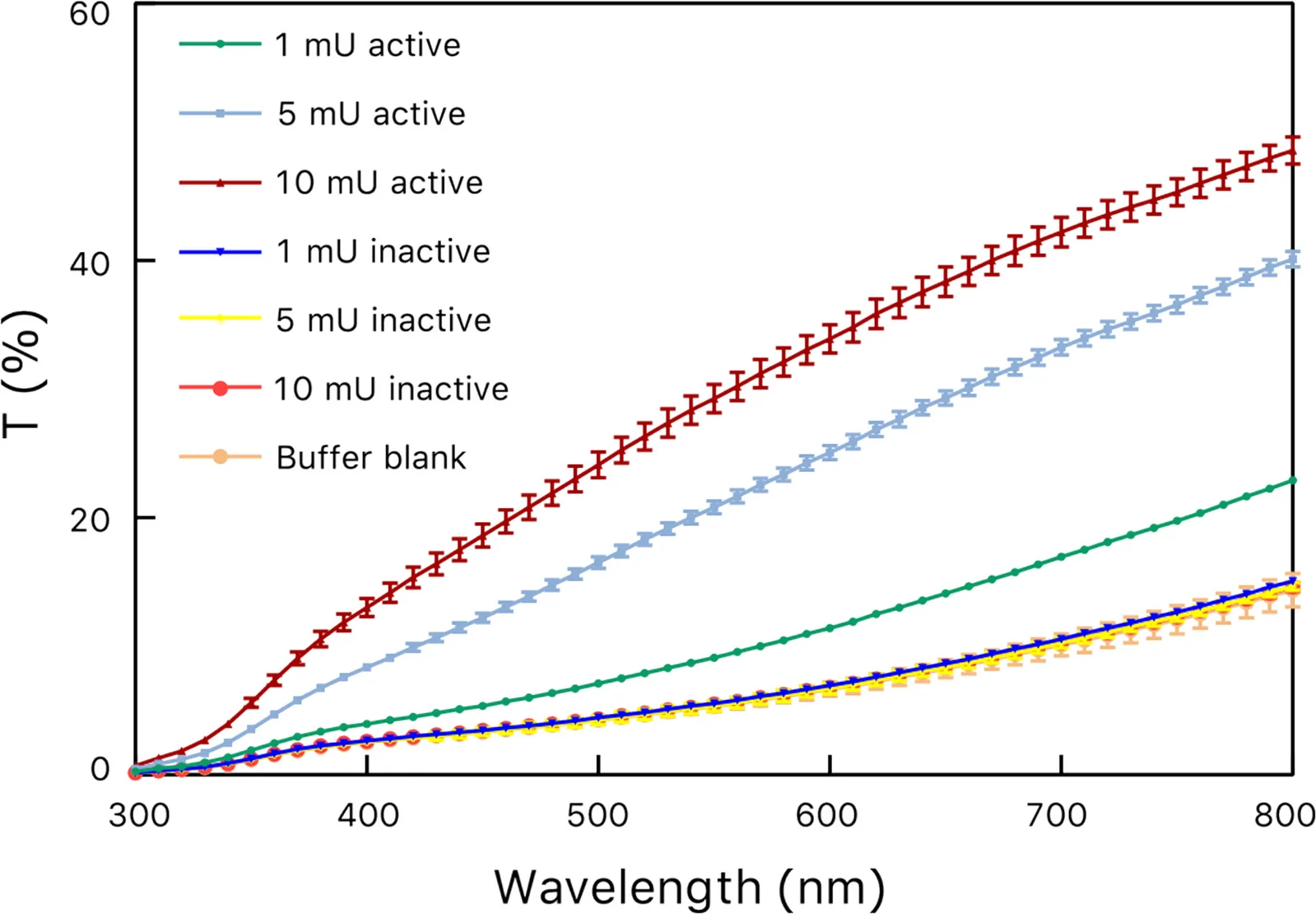
Transmittance spectra of the active wells (1, 5, 10 mU) and the matched blanks across 300–800 nm. Results are shown as means (n=12), with bars indicating standard deviations.

**Comparison of blank types.** To determine whether the heat-inactivated-enzyme blanks and the buffer-only blank could be treated as a single background, blank transmittance was modelled with a Bayesian linear mixed model (transmittance as a function of blank type and wavelength, with well included as a random intercept to account for the 51 repeated spectral measurements per well). Modelling the repeated measurements explicitly avoids the inflation of evidence that arises when correlated within-well readings are treated as independent [11]. Blank type had no material effect on transmittance: all four blank types lay within 0.15 %T of one another (95% credible interval 0.04–0.17 %T), a difference smaller than the assay detection limit expressed in transmittance units. The presence of heat-inactivated enzyme therefore did not meaningfully alter blank transmittance, and blank replicates were pooled for the estimation of background variability (σ_blank_).

**Final selection of the working wavelength.** For each wavelength, three quantities were derived from the group statistics, the blank-corrected signal (ΔT at 10 mU); the detection sensitivity (Ds) (slope S of ΔT versus activity divided by the blank standard deviation, S/σ_blank_); the response linearity (R² of ΔT against activity at 1, 5 and 10 mU). Two requirements were imposed, LOD (= 3.3 Ds^-1^) below 0.3 mU and a near-linear response (R² ≈ 0.98) across the working range; the violet region (< 440 nm) was excluded *a priori*, as the turbid gel drove transmittance towards the detector floor. Both requirements were met from the violet cut-off through approximately 510 nm (LOD ≈ 0.22–0.26 mU; R² ≈ 0.98), with signal and precision improving towards longer wavelengths. At 510 nm, at the long-wavelength edge of this window, sensitivity (LOD = 0.24 mU) was near maximal and linearity still well conditioned, so this wavelength was selected as the point of best overall performance (Fig. 2); the choice is robust, varying negligibly over ± 10 nm. The wavelength was fixed on the initial gel formulation and retained through the subsequent response-surface optimization, so all later development used a single wavelength; the 0.24 mU LOD reflects the initial substrate and is superseded by the definitive value on the optimized gel.

**Fig. 2:**
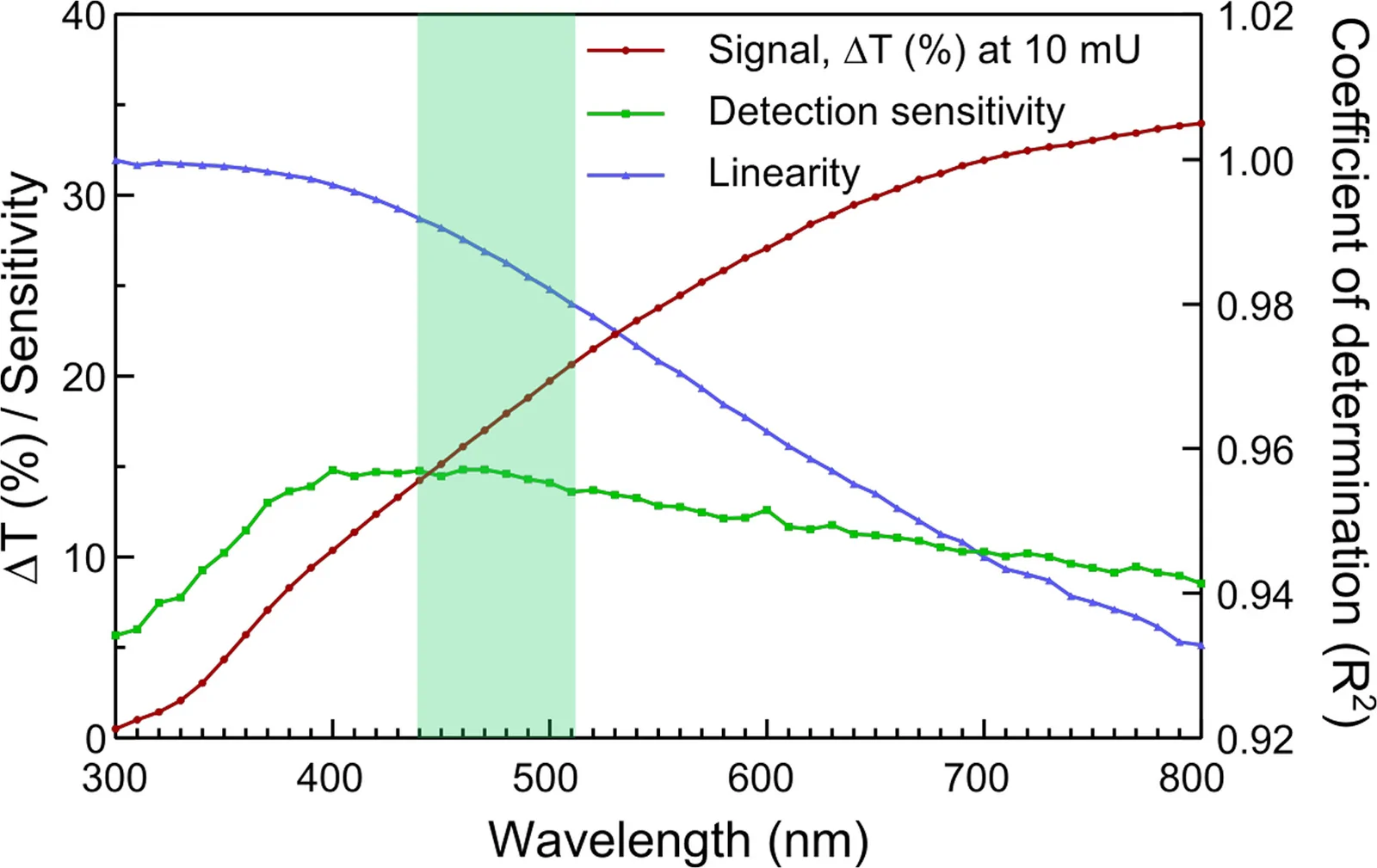
Wavelength dependence of the selection criteria: blank-corrected signal (ΔT at 10 mU), detection sensitivity (S/σ_blank_, inversely proportional to the LOD) and linearity (R² of ΔT versus activity, right axis). Signal improves toward longer wavelengths while linearity declines; the violet region (< 440 nm) was excluded a priori. The shaded region (≈440–510 nm) satisfies all criteria, and 510 nm was selected as its long-wavelength boundary.

**Substrate optimisation.** The composition of the zein-in-agarose substrate was optimised by response surface methodology using a three-factor, three-level Box–Behnken design (16 runs, including replicated centre points, n=3), varying agarose (0.50–1.50% w/v), zein (0.40–0.50% w/v) and well volume (100–200 µL). Three responses were modelled: calibration slope (sensitivity), calibration linearity (R²) and blank transmittance (summarised in Table 1). Sensitivity was maximal at an intermediate agarose concentration and decreased with zein and well volume (Fig. 3a); blank turbidity increased mainly with zein (Fig. 3b); and linearity depended on both agarose and zein but fit this response only weakly (Fig. 3c). Numerical desirability optimisation, maximising sensitivity and linearity while constraining the blank to an acceptable turbidity, identified an optimum of 1.21% agarose, 0.46% zein and 125 µL per well, which was confirmed experimentally and carried forward to method validation.

**Table 1:** Summary of the response-surface models for the three responses of the Box–Behnken design. Adeq. precision is the signal-to-noise ratio (>4 desirable); LoF, lack of fit. A, agarose; B, zein; C, well volume.

Response	Model	Model F (p)	R²	Adj R²	Pred R²	Adeq. prec.	LoF p	Significant terms
Slope (sensitivity)	reduced quadratic	31.6 (<0.0001)	0.940	0.911	0.820	19.3	0.127	A, B, C, A²
Blank (%T)	2FI	48.4 (<0.0001)	0.977	0.965	/	/	0.091	B, C, AC
Linearity (R²)	quadratic	7.55 (0.034)	0.944	0.819	/	9.6	0.375	A, B, C²

The lack-of-fit for the blank was marginally significant (p = 0.091); the blank model was used for guidance. The linearity model was down-weighted owing to its weaker fit.

**Fig. 3:**
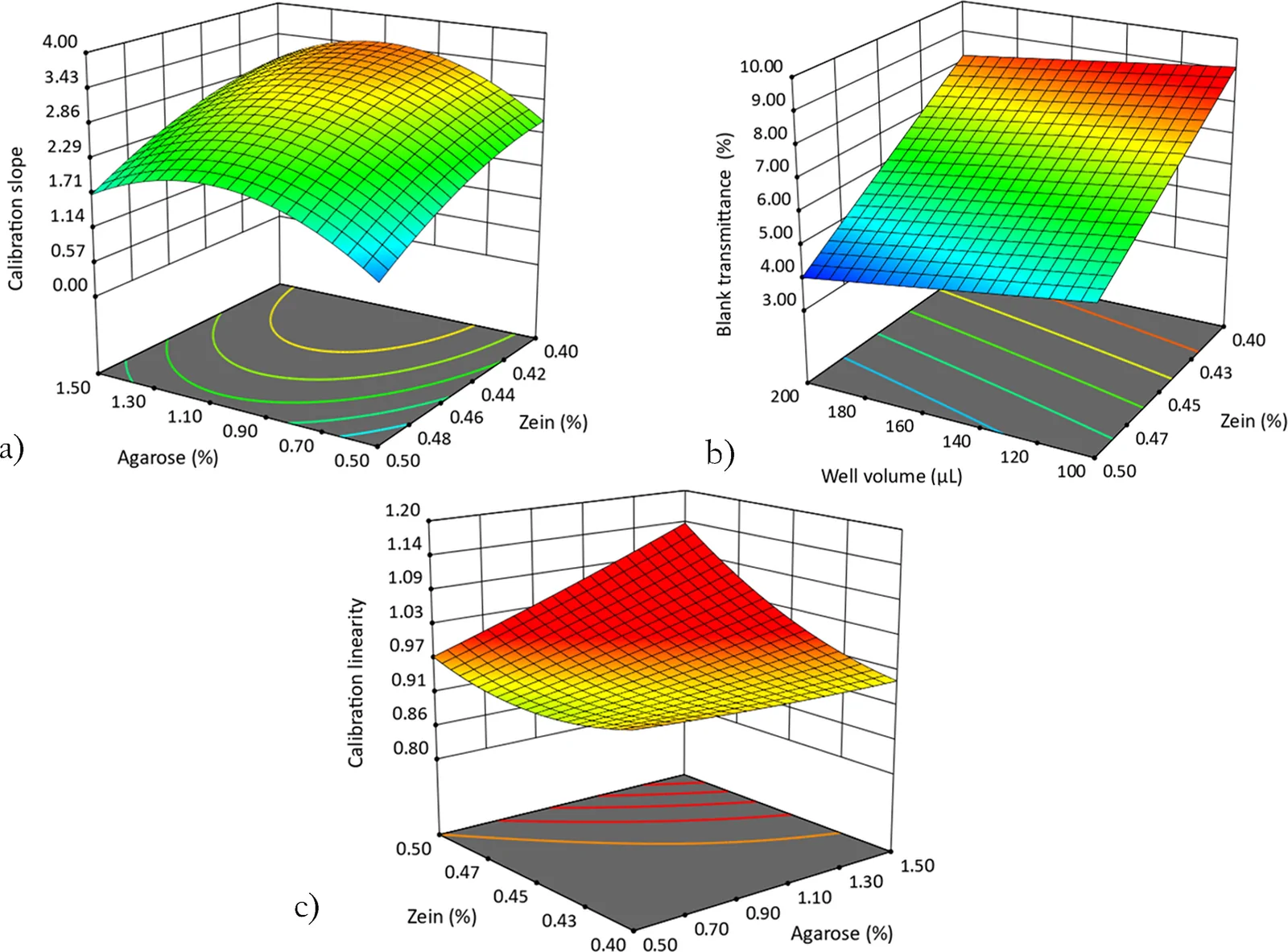
Response surfaces from the Box–Behnken design. (a) Calibration slope (sensitivity) as a function of agarose and zein concentration. (b) Blank transmittance as a function of zein concentration and well volume (agarose fixed at 1.21%). (c) Calibration linearity (R²) as a function of agarose and zein concentration (well volume fixed at 125 µL).

**Linearity and working range.** The optimum formulation was prepared and characterized over three independent days (independent gel batches and enzyme dilution series), with nine replicate (n=9) wells per activity level per day, using the fixed conditions established above (510 nm; 25 min incubation). Savinase standards spanned 0.1–10 mU per well. For each well the blank-corrected signal ΔT was computed against the same-day mean blank, and quantitation was performed against a same-day calibration; the pooled three-day data are reported here as the representative performance of the method.

Below 0.5 mU the net signal was indistinguishable from zero on two days (ΔT ≤ 0 at 0.1 and 0.25 mU), a lag consistent with the diffusion-limited onset of substrate clearing; this defined the lower bound of the assay, and the calibration was established over 0.5–10 mU.

The choice of calibration model was made explicitly rather than assumed. Both a straight-line and a second-order model were therefore fitted to the pooled three-day data and compared. Simple linear regression already gave a high coefficient of determination (R² = 0.985); however, three independent lines of evidence showed the linear model to be inadequate:

• *Formal lack-of-fit (Mandel) test.* Adding a quadratic term significantly improved the fit over the linear model (F(1, 153) = 157.7, p < 0.001; ΔR² = 0.007, from linear R² = 0.985 to quadratic R² = 0.993), and the quadratic coefficient was itself highly significant (β = −0.118 %T mU⁻², p < 0.001). The negative sign confirms the expected saturating curvature.
• *Structured residuals.* The residuals of the linear fit were not random but followed a systematic curved pattern, negative at both extremes and positive in the mid-range (−0.90, −0.72, +0.78, +1.69, +0.54 and −1.25 %T at 0.5, 1, 2.5, 5, 7.5 and 10 mU), which is the signature of unmodelled curvature. The second-order fit showed no such trend.
• *Accuracy of back-calculation.* Recovery of the calibration standards was poor and strongly range-dependent under the linear model (47.5% at 0.5 mU, ∼110% in the mid-range, 96% at 10 mU), whereas the second-order model recovered every level within ±6% (94.9–104.8%; Table 2).

**Table 2:** Accuracy of the calibration over the working range, comparing the linear and second-order models. Recovery is the back-calculated activity as a percentage of the nominal activity; ΔT is the mean blank-corrected transmittance (n ≈ 26 per level).

Nominal activity (mU)	Mean ΔT (%)	Linear recovery (%)ᵃ	Quadratic recovery (%)ᵇ
0.5	0.74	47.5	104.8
1	2.65	79.1	94.9
2.5	9.31	109.1	100.9
5	18.82	109.8	101.2
7.5	26.26	102.1	98.7
10	33.07	96.4	100.4

ᵃ Linear model: ΔT = 3.439 x − 0.072 (R² = 0.985). ᵇ Second-order model: ΔT = −0.118 x² + 4.647 x − 1.658 (R² = 0.993); Mandel test vs. linear model F(1, 153) = 157.7, p < 0.001.

A second-order calibration was accordingly adopted (Fig. 4). The pooled three-day calibration function was:

(4) ΔT=−0.118x²+4.647x−1.658

with the corresponding linear reference model

(5) ΔT=3.439x−0.072

**Fig. 4:**
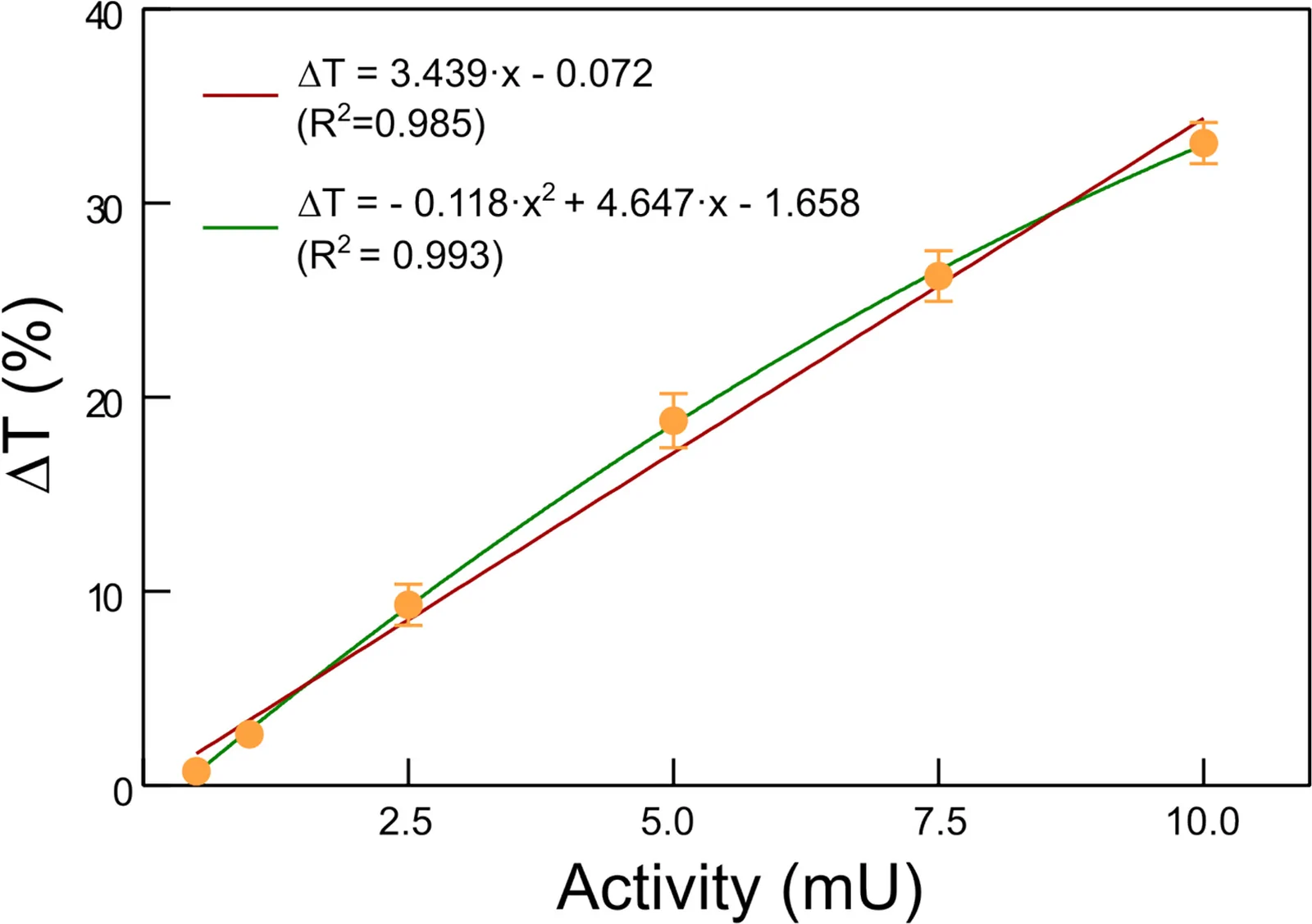
Calibration of the optimized assay (0.5–10 mU savinase, three days). The mean ± SD at each level are shown (n=26 per activity level), together with the adopted second-order fit and the linear reference model. The second order polynomial fit is indicated by the green line, and the linear fit by the red line.

In routine use, unknown activities are interpolated from a same-batch calibration of this form.

**Accuracy.** The quadratic model quantified all standards within the predefined acceptance range (85–115%), recovery, a standard bioanalytical criterion requiring back-calculated values to fall within ±15% of the nominal activity), whereas the linear model underestimated low activities and overestimated mid-range activities (Table 2).

**Limits of detection and quantification.** Detection limits were derived from the variability of the blank and the sensitivity of the calibration, as LOD = 3.3 Ds^-1^ and LOQ = 10 Ds^-1^. The blank was highly reproducible within each day (SD = 0.166, 0.120 and 0.138 %T on the three days; n = 22–24 per day), giving a pooled within-day σ_blank_ of 0.14 %T. This yields LOD = 0.10 mU and LOQ = 0.31 mU. The across-day standard deviation of the blank was substantially larger (0.40 %T), because the absolute blank level shifted between days (day means 6.83, 6.02 and 6.78 %T) but this is absorbed by the same-day calibration

*Note: this statistical LOQ is a theoretical lower bound; the empirically validated working range (established from day-to-day repeatability, below) begins at 1 mU, with 0.5 mU usable only for semi-quantitative purposes.*

**Precision.** Precision across the working range (Table 3) showed repeatability CV ≤13% from 1 mU upwards, falling to 2.4% at 10 mU; the higher apparent CV at 0.5 mU (36%) reflects the small net signal near the detection limit rather than poorer precision, since the raw transmittance CV remained low (2–3.5%) throughout. The assay is therefore reliable for quantitation over 1–10 mU, with 0.5 mU regarded as semi-quantitative. The high relative scatter at 0.5 mU reflects the CV's definition, not measurement imprecision: absolute ΔT scatter is roughly constant, but the mean net signal approaches zero near the detection limit, inflating the ratio. Raw transmittance CV stays low and stable (∼2–3.5%) throughout, confirming reproducible readings. The low-activity end is therefore not merely qualitative, though the precision data do not establish quantitation at 0.5 mU.

**Table 3:** Precision across the working range over three independent days (n = 8–9 wells per level per day; n ≈ 26 combined), as CV of the blank-corrected signal ΔT.

Nominal activity (mU)	Mean ΔT (%T)	Repeatability CV (%)ᵃ	Intermediate CV (%)ᵇ	Total CV (%)ᶜ
0.5	0.74	36	39	45
1	2.65	13	23	22
2.5	9.31	6.2	12.8	11.6
5	18.82	4.7	7.1	7.5
7.5	26.26	2.7	5.0	4.9
10	33.07	2.4	2.6	3.2

ᵃ Repeatability: mean of the three within-day CVs. ᵇ Intermediate precision: CV of the three daily mean signals (between-day component only). ᶜ Total: CV of all replicates pooled across days. For comparison, the CV of the raw transmittance was low and uniform across the range (3.0, 3.5, 3.5, 3.5, 2.2 and 2.0% at 0.5, 1, 2.5, 5, 7.5 and 10 mU), indicating stable measurement precision; the larger ΔT-CV at low activity reflects the small net signal there rather than reading imprecision.

**Incubation time.** Because the assay reads the endpoint of an ongoing digestion, incubation time governs how far the reaction has progressed and therefore the position of the quantification window. Calibrations at 20, 25, 30 and 40 min (Fig. S1) showed that longer incubation raises sensitivity and lowers the detection and quantification limits, while progressively curving the response as high-activity wells approach substrate exhaustion. Incubation time can thus be matched to the expected sample activity: short incubation (20 min) preserves a wide range for high-activity samples at low sensitivity, whereas long incubation (40 min) maximizes sensitivity for low-activity samples at the cost of the upper range.

**Screening-window quality (Z′-factor).** Suitability of the assay for hit identification was assessed by the Z′-factor [3], the standard screening-quality metric, on three independent days. On each plate, 48 wells received a full-signal positive control (5 mU savinase) and 48 wells a heat-inactivated negative control (blank), read as transmittance at 510 nm; four negative-control wells across the three plates were excluded as outliers (Grubbs, α = 0.05). The Z′-factor was calculated from the means (μ) and standard deviations (σ) of the two controls as:

(6) $$Z^{\prime }\;=\;1\;-\;\frac{3\left(\sigma_{+}\;+\;\sigma_{-}\right)}{\left|\mu_{+}\;-\;\mu_{-}\right|}$$

where the subscripts + and − denote the positive and negative controls. Both control populations were approximately normally distributed on each day (Shapiro–Wilk p > 0.05 for all but one group, p = 0.03 at n = 48), satisfying the assumption underlying the metric.

The positive control transmittance agreed with the value predicted for 5 mU by the calibration, confirming that the control lay within the validated working range (Table 4). The resulting Z′-factors were all comfortably above the 0.5 threshold that defines an excellent, screening-ready assay [3], and the separation window between the controls was large relative to their combined variability on every plate, indicating that active and inactive samples are reliably distinguished within a single run.

**Table 4:** Assay quality for screening across three independent days. Positive control, 5 mU savinase; negative control, heat-inactivated blank; transmittance at 510 nm (n = 48 per control per day, excluding outliers).

Day	Positive control	Negative control	Window (%T)	Z′
1	29.21 ± 1.33 (n = 48)	6.84 ± 0.16 (n = 45)	22.4	0.80
2	23.94 ± 1.74 (n = 48)	6.03 ± 0.14 (n = 47)	17.9	0.69
3	23.62 ± 1.29 (n = 48)	6.87 ± 0.17 (n = 48)	16.8	0.74
Mean	/	/	/	0.74 ± 0.06

**The sample matrix perturbs the background isolate-specifically.** On the zein-free gel the active samples and their heat-inactivated blanks were indistinguishable (ΔT scattered about zero), showing that no turbidimetric signal is generated in the absence of zein and that heat inactivation does not alter the sample's optical properties; the signal measured in the assay is therefore due to proteolysis of the zein substrate rather than to any change in the sample itself. The sample matrix is nevertheless not optically equivalent to buffer. On the zein-free gel it lowered transmittance relative to a buffer blank by 2.65 %T (Welch t = 11.9, p < 0.001), reflecting an intrinsic optical contribution of the pigmented matrix (light absorbance rather than scattering by a precipitate), and on the zein gel the heat-inactivated matched blanks were more turbid than a buffer blank by 1.98 %T (Welch t = 10.8, p < 0.001; 95% CI 1.62 to 2.35), a shift that combines this intrinsic contribution with a component dependent on the presence of zein (Fig. 5). The magnitude of this buffer-versus-matched-blank discrepancy was analysed across the six isolates by two-way ANOVA (isolate × substrate). The largest source of variation was the isolate itself (38.9 % of total variation, p < 0.001), exceeding both the zein/zein-free difference (15.3 %, p < 0.001) and their interaction (16.8 %, p < 0.001). In other words, the error introduced by replacing a matched blank with a common blank is not a fixed offset but varies from isolate to isolate, from about 0.1 to 2.9 %T (roughly 0 to 0.7 mU) across this small panel. Because this between-isolate component is the dominant term, it cannot be removed by any single shared blank or global correction; only a per-sample matched blank captures it.

**Fig. 5:**
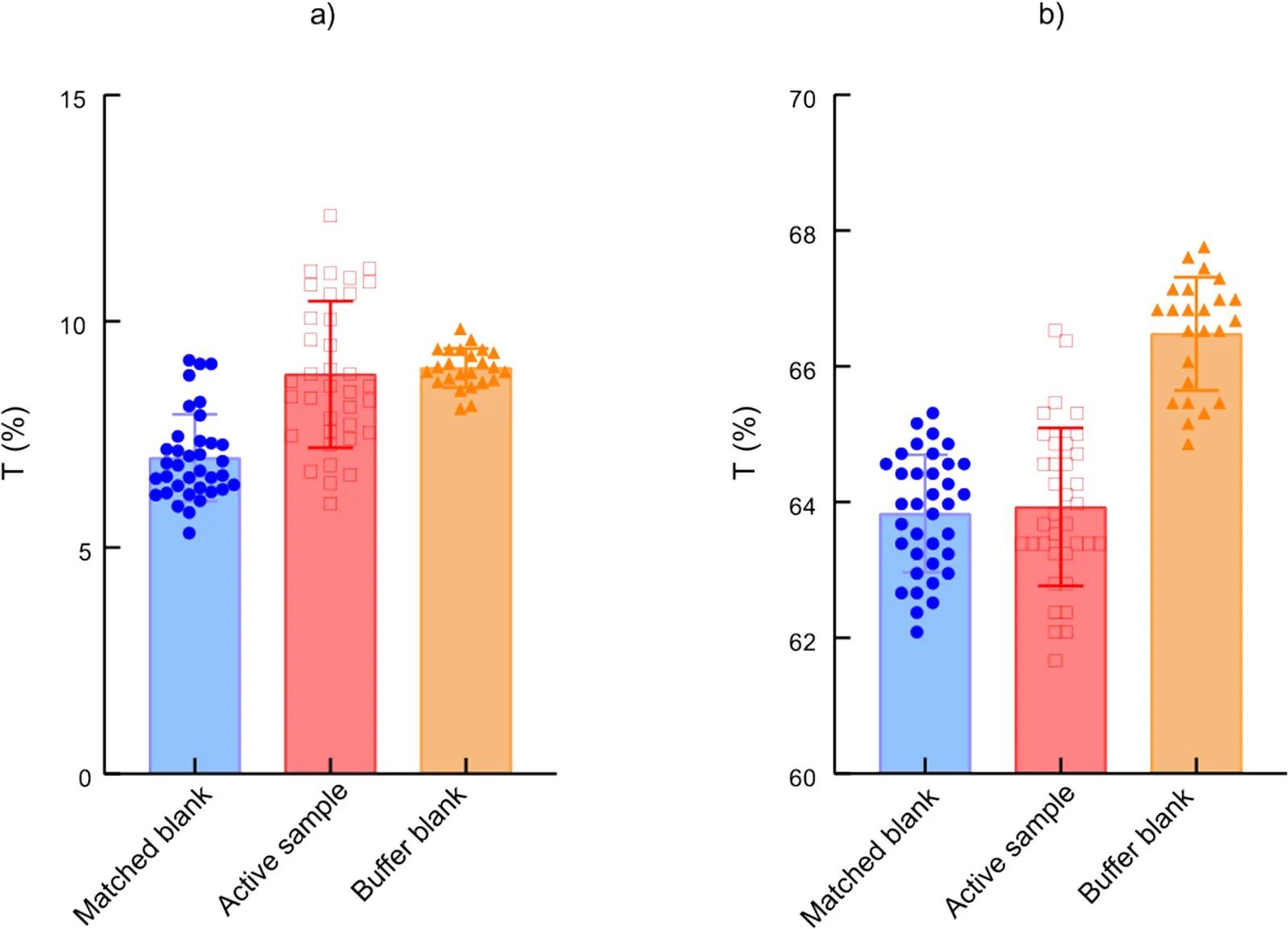
Effect of the cultured sample matrix on gel turbidity, measured as transmittance (%T) at 510 nm. Matched blank (heat-inactivated sample), active sample, and buffer blank are compared on (a) the standard zein-containing substrate and (b) a zein-free control gel (mean ± SD; individual replicates shown). On the zein gel (a) the matched blanks are more turbid than the buffer blank and vary between isolates, whereas on the zein-free gel (b) sample and matched blank are indistinguishable and only the buffer differs, showing that the sample matrix does not itself precipitate but alters the background turbidity through interaction with zein.

**Blanking strategies for larger sample numbers.** Table 5 summarises three blanking strategies and when each applies, from per-sample matched blanks (highest accuracy) to a single shared blank (only where the matrix is common across samples).

**Table 5:** Blanking strategies for larger sample numbers.

Strategy	When to use	What it corrects	Cost/limitation
Per-sample matched blank	Fine quantitation; heterogeneous panels; activities near the detection limit	Fully corrects isolate-specific background	+1 inactivated well and one heat-inactivation step per sample; scales poorly to large libraries
Shared matrix blank + conservative threshold	Primary screening of many isolates to flag active producers	Removes the mean matrix offset (∼2 %T); a 3.3 × SD threshold absorbs the residual variation	Blank must contain matrix (not buffer); bias is conservative (underestimates activity, no false positives)
Single shared blank	Sample sets with a common matrix (e.g. mutants from a single parent strain, one medium)	Negligible loss of accuracy when the matrix is uniform	Most economical option; must not be used on heterogeneous wild-type collections

For large screens these combine into a two-tier workflow: a primary screen with a shared matrix blank and a conservative threshold to flag candidates, followed by confirmation of the few hits with per-sample matched blanks, allowing the same assay to serve as both a scalable screen and a quantitative method.

## Limitations

The ZIMP assay specifically measures zein-degrading proteolysis and was validated against a single reference subtilisin (Savinase® 16L). Activities are therefore expressed as Savinase-equivalent units. Because the signal reflects the loss of acid-precipitable zein rather than the number of peptide bonds cleaved, enzymes with different cleavage patterns, for example limited proteolysis that leaves large acid-insoluble fragments, may be under- or overestimated relative to the reference. Proteases that do not cleave zein, and mechanistic classes other than subtilisins (aspartic, cysteine and metalloproteases), require separate verification, ideally against a class-matched reference enzyme.

Operating conditions are restricted to those validated: 50 mM Tris-HCl, pH 8.5, containing SDS, at 40°C. SDS may reduce the activity of detergent-sensitive proteases, and any other buffers, pH values or temperatures require revalidation of gel stability and calibration. ZIMP is an endpoint method: it does not report initial rates and is not suited to determining kinetic parameters on the insoluble substrate. Plates should be read within approximately 1 h of acid addition, before turbidity reaches saturation (data not shown).

The response is non-linear, so quantification requires a second-order polynomial calibration in each batch, and calibration should be repeated whenever the zein lot changes, as the composition of commercial zein may vary. The dynamic range is bounded at both ends. Below approximately 0.5 mU no net signal is generated within the 25 min incubation, consistent with the diffusion-limited onset of substrate clearing; activities between 0.5 and 1 mU are therefore semi-quantitative, and reliable quantification starts at 1 mU. At the upper end, the response approaches saturation as substrate is depleted, so samples exceeding 10 mU per well must be diluted into the working range, or the incubation time shortened (Table S1).

In pigmented or otherwise heterogeneous samples, such as crude fermentation broths, accurate quantification requires a heat-inactivated blank matched to each sample, which reduces the number of samples per plate. A shared blank is adequate only where the matrix is common across samples or where screening-level background uncertainty (approximately 0.5 mU) is acceptable. The matrix effect was characterised on a panel of six isolates only, and its magnitude may differ for other organisms or culture media. Likewise, the Z′-factor was determined with a purified reference enzyme in buffer; screening performance with crude samples, where matrix-dependent background adds to the variance of the negative control, is expected to be lower and should be verified for each sample type. Finally, the assay was validated in a single laboratory on one plate reader, inter-laboratory reproducibility has not yet been assessed, and activities obtained by ZIMP have not been compared directly with those from clearing-zone or casein-based assays on the same set of samples.

## Ethics statements

Not applicable. This method does not involve human subjects, animal experiments, or data collected from social media platforms.

## CRediT author statement

Nenad Drulović: Conceptualization, Methodology, Investigation, Data Curation, Visualization, Writing – Original Draft; Luka Blagojević: Conceptualization, Methodology, Investigation, Data Curation, Visualization, Writing – Original Draft; Aleksandra Margetić: Writing – Review & Editing; Nataša Božić: Writing – Review & Editing; Biljana Dojnov: Writing – Review & Editing, Supervision, Project administration, Funding acquisition; Zoran Vujčić: Supervision; Marinela Šokarda Slavić: Supervision, Visualization, Writing – Review & Editing.

## Related research article

From corn coproducts to bioactive peptides: an integrated bioprocessing approach

## Declaration of competing interest

The authors declare that they have no known competing financial interests or personal relationships that could have appeared to influence the work reported in this paper.

## Data Availability

Data will be made available on request.
