# Supplementary material for: Zein-in-agarose microplate turbidimetric assay for screening and quantifying protease activity
Source: MethodsX. 2026 Oct 19;17:104221. doi: 10.1016/j.mex.2026.104221 (PMC13735007; doi:10.1016/j.mex.2026.104221)
Supplement: Supplementary file 1 [file mmc1.docx]

**Table S1. Effect of incubation time on the calibration and quantification window.**

*Equations are quadratic fits (ΔT = a0 + a1x + a2x²) over the working range measured on the same day.*

| **Incubation** | **Calibration equation (ΔT)** | **adj R²** | **LOD (mU)** | **LOQ (mU)** | **Reliable range (mU)** |
| --- | --- | --- | --- | --- | --- |
| 20 min | −0.112 + 2.046x + 0.0167x² | 0.986 | 0.18 | 0.53 | 1–10 (low end erratic) |
| 25 min | −1.747 + 5.067x − 0.150x² | 0.998 | 0.10 | 0.31 | 1–10 (validated) |
| 30 min | −2.663 + 6.430x − 0.256x² | 0.999 | 0.08 | 0.25 | 1–10 |
| 40 min | −0.765 + 14.05x − 1.592x² | 0.991 | 0.03 | 0.10 | 1–5 |

Longer incubation therefore relocates the whole quantification window: it raises sensitivity and lowers the detection and quantification limits, while progressively curving the response as the high-activity wells approach substrate exhaustion. The incubation time can thus be chosen to match the expected activity of the samples. Short incubation (20 min) preserves a wide range for high-activity samples at low sensitivity, while long incubation (40 min) maximizes sensitivity for low-activity samples at the cost of the upper range.
